# A bespoke water T–maze apparatus and protocol: an optimized, reliable, and repeatable method for screening learning, memory, and executive functioning in laboratory mice

**DOI:** 10.3389/fnbeh.2024.1492327

**Published:** 2024-12-10

**Authors:** Jeremy Davidson Bailoo, Susan E. Bergeson, Igor Ponomarev, Joshua O. Willms, Brent R. Kisby, Gail A. Cornwall, Clinton C. MacDonald, J. Josh Lawrence, Vadivel Ganapathy, Sathish Sivaprakasam, Praneetha Panthagani, Scott Trasti, Justin A. Varholick, Michael Findlater, Amrika Deonarine

**Affiliations:** ^1^Department of Cell Biology and Biochemistry, Texas Tech University Health Sciences Center, Lubbock, TX, United States; ^2^Department of Pharmacology and Neuroscience, Texas Tech University Health Sciences Center, Lubbock, TX, United States; ^3^Garrison Institute on Aging, Texas Tech University Health Sciences Center, Lubbock, TX, United States; ^4^Department of Biology, University of Florida, Gainesville, FL, United States; ^5^Department of Chemistry and Biochemistry, University of California Merced, Merced, CA, United States; ^6^Department of Civil, Environmental, and Construction Engineering, Texas Tech University, Lubbock, TX, United States

**Keywords:** refinement, Morris water maze, MWM, T-water maze, water T-maze, reproducibility, protocol, animal welfare

## Abstract

The Morris Water Maze (MWM) is the most commonly used assay for evaluating learning and memory in laboratory mice. Despite its widespread use, contemporary reviews have highlighted substantial methodological variation in experimental protocols and that the associated testing procedures are acutely (each trial) and chronically (testing across days) stressful; stress impairs attention, memory consolidation and the retrieval of learned information. Moreover, the interpretation of behavior within the MWM is often difficult because of wall hugging, non-spatial swim strategies, floating, and jumping off the escape platform. Together, these issues may compromise the reproducibility, generalizability, and predictability of experimental results, as well as animal welfare. To address these issues, and as an initial proof-of-principle, we first narrowed the spatial dimensions of the MWM by using a T-insert, which constrained and reduced the overall length of time/distance that the animal must swim in order to navigate to the escape platform, thus reducing stress and off-task behavior. Given the robust performance observed across spatial acquisition (learning and memory) as well as during reversal learning (executive function), we further reduced (by 43%) the overall distance and time that the animal must swim in order to find the escape platform in a bespoke standalone Water T-Maze (WTM). We show, across five experiments, procedural refinements to our protocol and demonstrate robust, reliable and reproducible indicators of learning, memory and executive functioning in a task that is also significantly more efficient (3 days of testing within the WTM vs. 11 days of testing within the MWM). Taken together, our WTM apparatus and protocol are a significant improvement over other water-based apparatuses and protocols for evaluating learning, memory, and executive functioning in laboratory mice.

## 1 Introduction

Poor reproducibility in preclinical research, the so-called reproducibility crisis, is often attributed to poor experimental design and conduct and poor reporting of experimental detail in published research (Bailoo et al., 2014; Ioannidis, 2005; Ioannidis, 2006; Baker, 2016). Far less emphasis has been placed on investigating the effects of seemingly innocuous variables that might significantly affect the measurement and reproducibility of behavioral and other biomarkers (e.g., standards for housing and care, social behavior within the home-cage, animal welfare) (Bailoo et al., 2010; Bailoo et al., 2018; Bohlen et al., 2014; Wahlsten, 2010a; D'Hooge and Deyn, 2001; Sorge et al., 2014; Kafkafi et al., 2003; Kafkafi et al., 2005; Crabbe et al., 1999; Wahlsten et al., 2006; Arroyo-Araujo et al., 2022; Martin-Arenas and Pintado, 2014; Chacon-Teran et al., 2023; Liu et al., 2024; Gulinello et al., 2019; Schellinck et al., 2010; Sare et al., 2021; Richter et al., 2011; Mandillo et al., 2008; Gygax et al., 2024; Varholick et al., 2019; Varholick et al., 2018; Dutton et al., 2018; Bennett et al., 2018). This is especially true for the Morris Water Maze (MWM)—the most commonly used task for the assessment of learning, memory and executive function—in the most common preclinical animal model, *Mus musculus* (Wahlsten, 2010a; D'Hooge and Deyn, 2001; Lang et al., 2023; Macri and Richter, 2015; Malakoff, 2000; Wahlsten, 2010b; Kapadia et al., 2016).

The MWM task was first described in 1981, and later formalized as a protocol in 1984, as a means of evaluating spatial acquisition (learning and memory) in the laboratory rat (Morris, 1981; Morris, 1984). Since then, the MWM protocol has been adapted to evaluate reversal learning (executive functioning), repeated learning, discrimination learning, latent learning and cued learning; spatial acquisition and reversal learning remain the most commonly evaluated behavioral outcomes, however (Vorhees and Williams, 2006; Brandeis et al., 1989; Brown and Tait, 2010). Spatial acquisition training involves placing the animals at one of four pre-determined start locations and recording the latency to swim to a hidden escape platform or the distance traveled within the maze. Importantly, only one of these measures should be reported to avoid issues of pseudoreplication, as both measures are generally significantly and positively correlated (Wahlsten, 2010a; Colegrave and Ruxton, 2018; Lazic, 2010). Generally, four trials are given per day, allowing for the counterbalancing of the four start locations (Vorhees and Williams, 2006; Nunez, 2008). A trial limit of two minutes for rats, and one minute for mice, with an inter-trial interval (ITI) of 60 s is common. Spatial acquisition training occurs across five days; a probe trial, which evaluates the strength of the learned spatial association, generally occurs on a separate day, day six, as a single 30 s trial (Vorhees and Williams, 2006). Reversal learning, where the escape platform is moved to a new location, can be completed within a further five days of testing (Vorhees and Williams, 2006). Thus, 11 days of testing per animal are required to evaluate spatial acquisition and reversal learning in the MWM.

With the advent of gene targeting technology, the MWM protocol was subsequently used with mice, often in a generalized behavioral screening test battery for newly generated genetically modified mice (Wahlsten, 2010a; Crawley and Paylor, 1997) or as part of a behavioral test battery investigating deficits in learning and memory (Voikar et al., 2001; Leighty et al., 2004; Iida et al., 1999). Despite being the most common behavioral screening tool for the study of spatial learning and memory in mice, numerous reviews and protocols have highlighted significant variation in the operationalization and execution of the MWM protocol and, in turn, the validity of the derived experimental results (Wahlsten, 2010a; D'Hooge and Deyn, 2001; Kapadia et al., 2016; Vorhees and Williams, 2006; Brandeis et al., 1989). These factors range from the physical features of the apparatus and the extra-maze environment to the protocol itself. One systematic review identified 38 task parameters that were unsystematically varied across studies and, of which, five explained 33–59% of the variation in commonly measured primary outcome variables, e.g., distance traveled, latency to platform, swim speed, time in quadrant (Wahlsten, 2010b). These results, as well as our own work, which has demonstrated the poor precision of automated tracking within water-based maze tasks (Bailoo et al., 2010), highlight the unmet need for refinement of the MWM apparatus and protocol.

The interpretation of behavioral data derived from the MWM is often difficult because of various strategies adopted by mice when placed within the apparatus. For example, mice commonly display thigmotaxic and circling behavior, swimming from and returning to the periphery of the maze, which results in long training times and an increased probability that animals may not learn to find the platform (Wahlsten, 2010a; D'Hooge and Deyn, 2001; Vorhees and Williams, 2006; Brandeis et al., 1989). Mice can also float passively, jump off the escape platform once reached and continue to swim (off-task behavior), or use non-spatial strategies (e.g., *praxis* and *taxis* strategies) to locate the platform. These off-task strategies have been shown to account for significantly more variation than the spatial memory factor that the MWM protocol is purported to measure in various mouse strains (Wolfer et al., 1998). Poor understanding of the task demands within the MWM leads to long training times and results in high levels of experienced stress (Francis et al., 1995; Harrison et al., 2009; Holscher, 1999). Stress, in turn, has been shown to impair attention, memory consolidation and the retrieval of learned information, rendering the consequentially obtained results questionable (Kapadia et al., 2016; Francis et al., 1995; Holscher, 1999; Sandi and Pinelo-Nava, 2007).

The primary physiological stressor associated with the MWM protocol is the inability of mice to effectively thermoregulate when placed into water where the temperature is below its thermoneutral zone, i.e., < 30°C, resulting in hypothermia (Vorhees and Williams, 2006; Nunez, 2008; David et al., 2013; Speakman and Keijer, 2012; Overton, 2010; Iivonen et al., 2003; Roder et al., 1996). Across studies, the water temperature within the MWM can range from 15 to 27°C, often without mention of whether or how the water temperature is maintained within a test session (Wahlsten, 2010b). Experimentally, it has been shown that swimming in 20°C (e.g., unheated water from a laboratory faucet) for 45 s trials, the average trial length in the MWM, is sufficient to cause a core body temperature decrease of 9°C, and correspondingly, a significant decrease in swim speed (Iivonen et al., 2003). These confounding effects can be minimized by increasing the water temperature to 24–26°C and increasing the ITI to 13 min (Wahlsten, 2010b; Iivonen et al., 2003). While these recommendations are sound and based on a well-grounded theoretical knowledge of mouse biology, they also render moot the standard MWM protocol as a quick means of screening for deficits in learning, memory and executive function.

Here, we establish an improved apparatus design over the existing MWM, a bespoke standalone water T-Maze (WTM), and develop and validate a protocol for spatial acquisition and reversal learning that addresses the above-listed issues with the MWM in non-impaired mice. Our refinements to the MWM make use of over a decade of experience with mouse behavioral testing while our synergistic research practices incorporate aspects of mammalian biology, animal welfare, and experimental design with a focus on reliability and reproducibility (Bailoo et al., 2010; Bailoo et al., 2018; Bohlen et al., 2014; Liu et al., 2024; Varholick et al., 2019; Varholick et al., 2018; Bailoo et al., 2014; Bailoo et al., 2018; Bailoo et al., 2016; Bailoo et al., 2020; Hintze et al., 2018). It should be noted, however, that while any proposed refinements to the MWM protocol should reduce the number and severity of stressors, it is impossible to completely remove experienced stress, as learning to escape in water-based mazes is predicated upon water-aversion-related-motivated behavior (D'Hooge and Deyn, 2001).

We employed a stepwise process to our refinements and detail them here to avoid unnecessary experimental duplication and animal use. Our overarching goals were: (1) to reduce-off task behavior and the use of non-spatial strategies to escape the maze; (2) to reduce experienced stress by minimizing the amount of time the animal spends in water below its thermoneutral range, and (3) to simultaneously develop a protocol that was methodologically efficient to reduce overall testing times.

## 2 Materials and methods

### 2.1 Mouse husbandry and housing

All mice used in the following experiments were maintained within the vivarium located at the Laboratory Animal Resources Center (LARC) at the Texas Tech University Health Sciences Center (TTUHSC), Lubbock Campus. Animals were kept on 14/10 light cycle, with lights off at 18:00 h. All mice were provided with *ad libitum* access to food (LabDiet^®^ 5R53) and hyperchlorinated tap water treated using reverse osmosis.

Two weeks prior to testing, animals were individually housed within Tecniplast GM500 IVC cages, as aggressive behavior between male mice is commonly observed when group housed animals are reunited subsequent to testing. During this two-week period, animals were extensively handled as tail-handling has been shown to induce high levels of stress and anxiety and negatively impacts learning and memory performance (Bailoo et al., 2018; Bailoo et al., 2018; Bailoo et al., 2020; Hintze et al., 2018; Novak et al., 2015; Gouveia and Hurst, 2013; Gouveia and Hurst, 2017; Gouveia and Hurst, 2019; Gerlai, 1999). Briefly, handling consisted of gently scooping the animal into the palm of the hand and allowing it to explore before returning it to the cage. A cage mate was then scooped, and the procedure repeated, with a maximum of 3–5 scoops per session. In the initial handling sessions, mice were sometimes cupped (picked up between closed hands) and then transitioned to scooping once the animal no longer jumped away. An animal was considered ready for testing if it voluntarily stayed within the experimenter’s hand for at least 10 s.

### 2.2 Mice

The mice used in all experiments were derived from collaborative pilot experiments with J. D. Bailoo which involved testing various genetic mutants or modeling diseased states; only data from control/wildtype animals are presented here (Table 1). The projects from which these control/wildtype animal data are derived were approved by the Institutional Animal Care and Use Committee at TTUHSC. Those projects were conducted in accordance with the local legislation, federal guidelines, and institutional requirements.

**Table 1:** Description of mice by strain, replicate, sex and age across experiments.

Experiment	Strain	Replicate	# Males	# Females	Total (n)	Age (months)
1	B6STOCKF1.Cg-Gt(ROSA)26Sor^tm14(CAG-tdTomato)Hze^, Sst^tm2.1(cre)Zjh^/JL	N/A	3	6	9	4–5
2	C57BL/6NCrl	N/A	5	0	5	2–3
3	C57BL/6NCrl	N/A	7	0	7	2–3
4	C57BL/6J	N/A	5	4	9	9–10
	J:ARC(S)	N/A	4	4	8	10–11
5 and 6	C57BL/6J	1*	5	4	9	9–10
		2	4	6	10	9–10
		3	2	2	4	9–10
		4	3	4	7	9–10
		5	8	7	15	9–10
	J:ARC(S)	1*	4	4	8	10–11
		2	4	4	8	10–11
		3	4	4	8	10–11
		4	4	4	8	10–11
		5	7	6	13	10–11

^*^These are the same animals as Experiment 4.

In these collaborative experiments, animals were always tested in groups of four and counterbalanced for sex and treatment (treatment/control or knockout, mutant/wildtype). Thus, one male and one female control/wildtype mouse, that is, the mice used in this paper, were always represented within a block of four mice. The experimenter was blinded to treatment allocation during testing.

### 2.3 Apparatus

#### 2.3.1 Experiment 1

**Figure 1:**
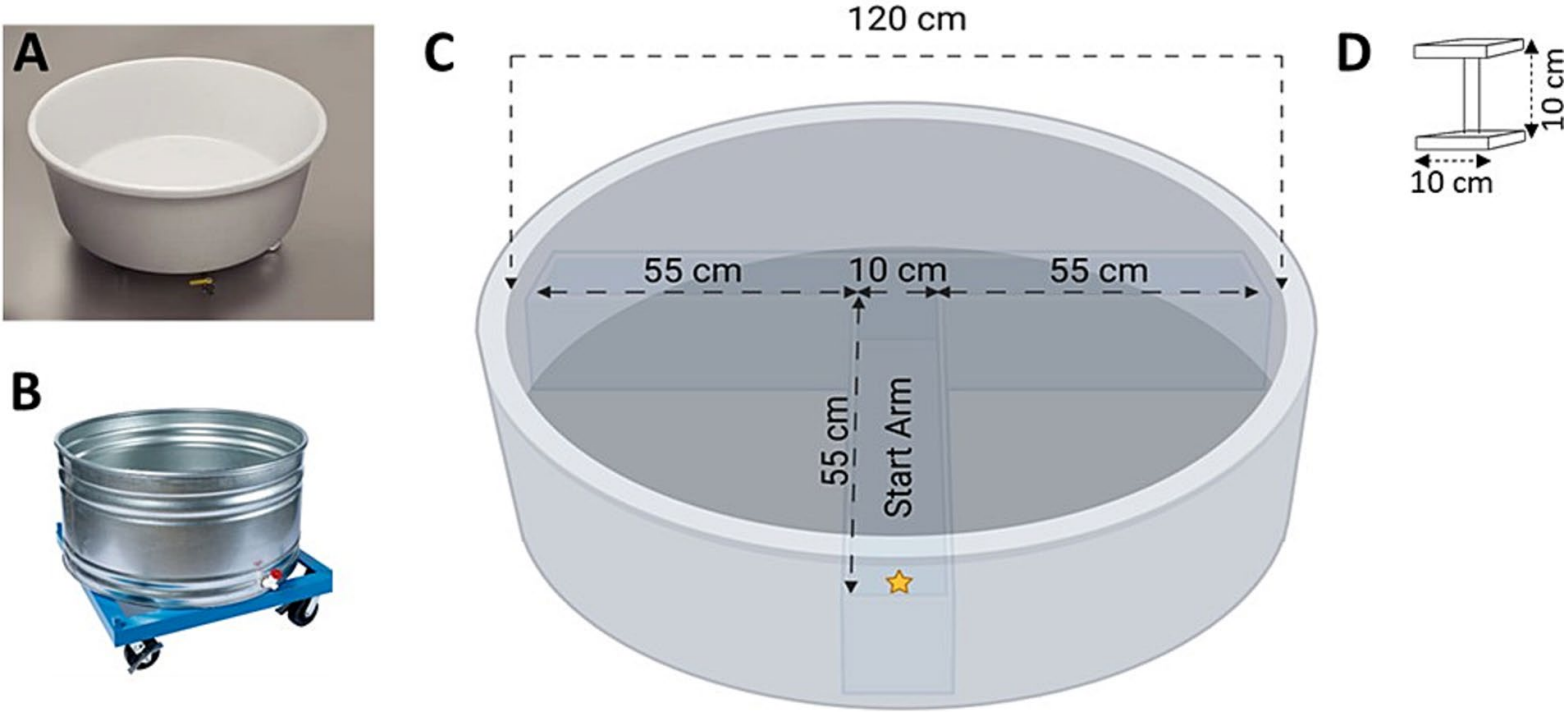
**(A)** Example of a Morris Water-Maze sold by Noldus (Ethovision XT video tracking); **(B)** Example of a Morris Water Maze sold by Stoelting (ANY-Maze video tracking); **(C)** Top-view schematic of the T-insert within the Morris Water Maze; **(D)** The escape platform. Schematics in **(C,D)** not drawn to scale (created with BioRender.com).

In a standard MWM protocol, on each day of testing, animals are started from four randomized locations within the apparatus and have to use spatial cues within the room to locate a hidden platform to escape (Vorhees and Williams, 2006). However, many of the common variants of the MWM apparatus are made from opaque materials and filled with water to a height which prevents the animal from being able to map distal room cues in order to spatially navigate to the hidden escape platform (Figures 1A,B; Wahlsten, 2010b; Vorhees and Williams, 2006). These issues, coupled with the thigmotaxic and circling behavior and subsequent waterlogging of the animal, may result in experimental data that are artefactual and idiosyncratic.

To eliminate these issues, J. D. Bailoo designed a bespoke MWM, 120 cm in diameter, made of clear acrylic, which permitted the animals to easily observe distal and proximal extra-maze room cues. The walls were 0.64 cm thick while the base was 1.27 cm thick to accommodate the weight of the water when filled. The walls were chemically welded to the base to ensure water tightness. A T-insert made of clear acrylic was used to narrow the swimmable spatial dimensions within the MWM. The walls of the T-insert were 0.64 cm thick, with each arm 55 cm in length and the center square 10 cm in length (Figures 1B,C). The square platform was also made of clear acrylic, with a platform area of 100 cm^2^ (10 × 10 cm) and a height of 10 cm. Both the MWM and T-Insert were built by Maze Engineers and housed at the Garrison Institute of Aging (GIA) behavioral facility at TTUHSC.

Animals were placed within the start arm (Figure 1C, location designated by a yellow star) and allowed to explore the maze to locate the hidden escape platform either located in the left or right arm. The maximum trial duration was 60 s; if the animal did not locate the platform within 60 s, it was placed onto the platform at the end of the first trial. On the first trial only, all animals remained on the platform for 30 s before being returned to its pre-warmed holding cage or forced air warming chamber (see Section 2.4.1). We predicted that by reducing the swimmable area to the “T,” off-task behavior such as swimming in concentric circles would be eliminated, making trial lengths shorter and improving learning performance (i.e., the latency to the escape platform) across simple discrimination (i.e., spatial acquisition) and reversal (i.e., executive functioning). Given our prediction of significantly shorter trial lengths, as well as our implementation of strategies to reduce thermoregulatory distress (see Section 2.4.1) a fixed ITI was not used; animals were tested immediately after the previous animal was tested.

#### 2.3.2 Experiments 2–6

**Figure 2:**
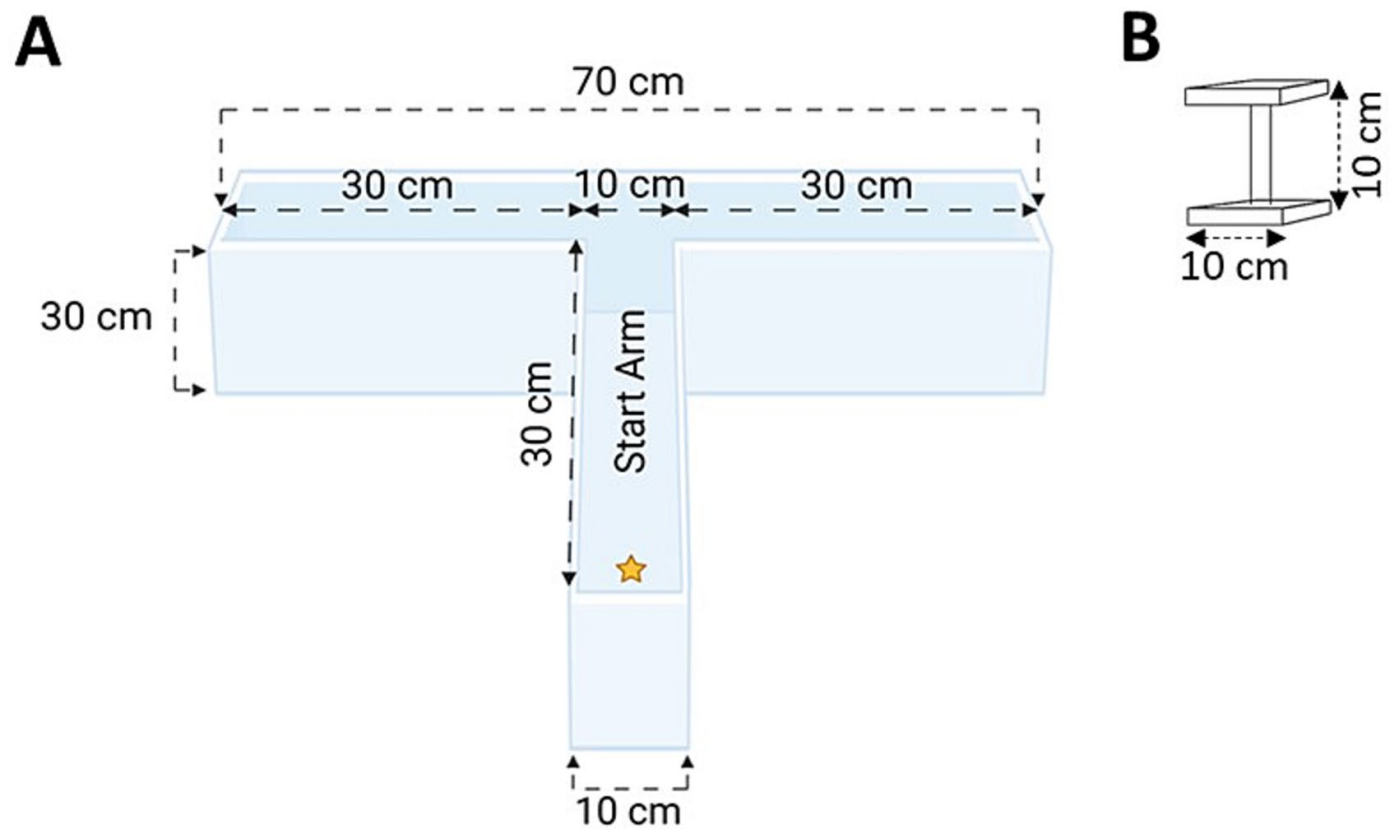
**(A)** Top-view schematic of our WTM; **(B)** The escape platform. Schematics not drawn to scale (created with BioRender.com).

Given the robust performance observed in both spatial acquisition and reversal learning in Experiment 1, in Experiment 2 we first explored whether experienced stress could be further minimized. To accomplish this, we further reduced the distance that the animal had to swim in order to find the escape platform by 43% (from 120 to 70 cm), based on recommendations in the previous literature (Wahlsten, 2010b). Here, J. D. Bailoo designed, and with the assistance of A. Deonarine, built a bespoke, standalone WTM made of clear acrylic (Figure 2). The walls and base of the WTM were 0.95 cm thick to accommodate the weight of the water when filled. The walls were chemically welded to the base to ensure water tightness. The arms of the WTM were aligned and then chemically welded (fused) together such that there were no seams; this eliminated off-task behavior where mice sometimes attempt to climb out of the maze when seams are present at joints in the apparatus (Vorhees and Williams, 2006). Using the same general procedures described in Experiment 1, we observed robust learning performance within our WTM, in both simple discrimination and reversal. This apparatus and these general procedures were used to further refine and validate our experimental protocol in Experiments 3–6.

#### 2.3.3 Infrared backlighting

In all experiments, the apparatuses were placed upon bespoke infrared emitting (850 nm) lightboxes, designed by J.D. Bailoo, and built with the assistance of A. Deonarine, to improve the precision of automated behavioral tracking (c.f., Bailoo et al., 2010). A bespoke universal serial bus (USB) powered camera, with a Sony IMX322 sensor, a 2.8–12 mm varifocal lens and an attached infrared pass filter (850 nm), was centered over the apparatus. The water level within both apparatuses was filled to a height of 11 cm (i.e., 1 cm higher than the platform) and rendered opaque using BLICK™ Essentials white non-toxic tempura paint (Item#:00057–1009). The animal’s behavior was automatically tracked using Noldus Ethovision XT v14 in Experiment 1 and with Stoelting’s ANY-Maze v6 in Experiments 2–6 using our previously established methods (Bailoo et al., 2010).

### 2.4 Procedures

#### 2.4.1 Methods used to decrease thermoregulatory distress

In Experiment 1, each animal was removed from its home-cage and individually placed into a holding cage (i.e., a Tecniplast GM500 cage) lined with three paper towels during the testing period when the animal was not in the maze. These holding cages were placed upon a warming pad set to 30°C (i.e., thermoneutral; Overton, 2010; David et al., 2013). In Experiments 2–6, when the animal was not in the maze, it was removed from its home-cage and individually placed into one of the four chambers of a forced air warming unit (Datesand Group Mini Thermacage) that was maintained at 30°C during the testing period. All holding cages/chambers were thoroughly dried and cleaned with 70% isopropyl alcohol after testing of each group of four animals was completed. The paper towels used in the holding cages were also replaced.

In all experiments, the water temperature within the apparatus was maintained between 25 and 26°C, slightly below thermoneutrality, to motivate the animals to swim to the hidden escape platform (David et al., 2013; Overton, 2010; Iivonen et al., 2003). Here, the water temperature was measured 1 cm from the water surface at the start, in both the left and right arms before each group of four animals were tested. If the water temperature decreased below this range, the water was heated using a 1,500-watt immersion heater. If the water temperature range was surpassed (i.e., warmer), mixing with the water at lower depths using the unplugged immersion heater was sufficient to achieve the prescribed temperature range.

#### 2.4.2 Habituation to the local test environment

Animals used in Experiment 1 were transported from the LARC and tested within the behavioral facility at the TTUHSC GIA behavioral facility. All of the remaining experiments were conducted within the LARC. For all experiments, animals were brought from their housing room to a holding area adjacent to the testing room 1 h before testing (i.e., at 17:00), as a means of habituation to the local test environment. At 18:00 (the start of the dark cycle), the light in the holding area was switched off and a Phillips PAR 38 red light emitting diode (LED) flood light was turned on. For behavioral testing, animals were brought from the holding room into the testing room, which was illuminated by a 40-lux white fluorescent light, to minimize anxiety-like behavior (Martin-Arenas and Pintado, 2014; Chapillon and Debouzie, 2000). This light was located to the left of the apparatus and served as a distal spatial cue.

#### 2.4.3 Protocol development and rationale

Our protocol in Experiment 1 was modeled after the MWM. The T-Insert was placed within the MWM which narrowed the swimmable area to within the “T” (Figure 1C); this served to reduce off-task behavior and to minimize the use of non-spatial strategies to escape the maze. The animal was released from the start arm (location designated by yellow star, Figure 1C). The escape platform was placed in the left arm for simple discrimination (i.e., spatial acquisition) during 3 days of training with four trials per day. On Day 4, the first trial for that day (trial 13) was used as a recall trial of the previously learned response. Thereafter (i.e., from trial 14), the platform was moved to the right side of the apparatus and an operationalized aspect of executive functioning (i.e., reversal learning) was evaluated next.

In Experiment 2, we further reduced the distance from the start location to the escape platform by 43% in our WTM. The associated protocol in Experiment 2, was also modeled after the MWM, with 5 trials per day across 4 days of testing. The animal was released from the start arm (location designated by yellow star, Figure 2) and the escape platform was placed in the left arm for simple discrimination (days 1–2 of testing). On Day 3 (trial 11), the location of the escape platform was switched to the right side of the apparatus for evaluation of reversal learning (days 3–4 of testing).

Given that the average trial length observed within our WTM in Experiment 2 was significantly shorter than the average trial length observed when using the T-insert within the MWM, we hypothesized that our protocol could be further modified by increasing the number of trials per day from five to 10 and decreasing the overall number of days of testing from four to three. To evaluate the reliability of this modified protocol, in Experiment 3 animals completed three additional sessions of reversal learning, where the platform location was alternated between the left and right arms across the 10 trials of each session.

In Experiment 3, we noted that during simple discrimination only, animals may become waterlogged when performing 10 trials per day. In addition, asymptotic learning performance across serial reversals was already observed by trial 6 of each stage. These two observations highlighted another opportunity for protocol refinement, where the number of trials could be decreased from 10 to 6 trials per day and where simple discrimination and reversal learning testing could be completed across 3 days (nine trials per stage). Additionally, as Experiments 2 and 3 made use of only male mice, we further extended our evaluations to both sexes, in a common inbred and outbred strain of mouse, respectively.

In Experiment 4, animals displayed a robust learning response across simple discrimination and reversal, with no evidence of waterlogging. We next evaluated the generalizability and replicability of this finalized protocol in Experiment 5 by testing 4 additional replicates of male and female inbred mice and outbred mice, respectively.

**Figure 3:**
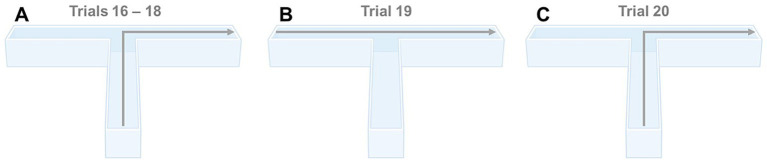
**(A)** Predicted behavioral response when released from the start arm in the WTM during the last 3 trials of reversal learning (trials 16–18); **(B)** Predicted behavioral response when released from the left arm in the WTM (trial 19); **(C)** Predicted behavioral response when again released from the start arm in the WTM (trial 20).

It has previously been reported that mice in multi-arm-water-based apparatuses may use non-spatial strategies to locate the platform in order to escape (D'Hooge and Deyn, 2001). For example, animals may either employ a sequence of movements to approach the platform (praxis strategy), use proximal cues to locate the platform (taxis strategy), or use distal cues within the spatial configuration of the apparatus (mapping/spatial strategy) (D'Hooge and Deyn, 2001). We predicted that animals would use a mapping strategy, attending to distal cues in order to locate the hidden platform within our WTM. We evaluated this prediction in Experiment 6. Here, the animals used in Experiment 5 performed two extra trials (trial 19 and 20) at the end of reversal learning, where the platform was located in the right arm. On the first trial, the animal was released from the left arm (instead of the start arm) while in the second trial, the animal was released from the start arm. If the animal employed a taxis strategy, it should swim into the start arm (trial 19) or to the right arm (trial 20). However, if as predicted a spatial strategy was used, the animal should swim directly to the escape platform, regardless of the start location (c.f., Figure 3).

### 2.5 Data checks and statistical analyses

Prior to analysis, the precision of automated tracking in each experiment was verified using our previously described methods (Bailoo et al., 2010). We also evaluated whether mice consistently displayed side-biased responses (e.g., always swam to the right arm); no side-biased responding was observed. Our primary outcome variable in all experiments was the latency of the animal to the escape platform on each trial. We predicted that the latency to the escape platform would be longer in the initial trials, during learning, and shorter in the later trials, once the animal had learned to swim to the escape platform in order to be removed from the water. We therefore hypothesized that a logarithmic function would best describe the predicted relationship between our outcome variable and trials within a stage (i.e., simple discrimination or reversal). To evaluate this predicted relationship, a log-linear curve fitting regression approach was used in IBM SPSS v29. Sex was included as a control variable, where applicable (Experiments 1, 4, and 5). Stages, i.e., simple discrimination or reversal learning, and mouse strains (Experiments 4 and 5) were analyzed separately. In order to evaluate the strength of learning, we used a one-sided directional paired samples t-test to compare: (1) the latency to the escape platform between the first and last trial of each stage and (2) the latency to the escape platform between the last trial and first trial of each stage. To evaluate the confounding effects of waterlogging, we also used a paired samples t-test to compare the swim speed between (1) the first and last trial within a day and (2) trials when the stage was switched from simple discrimination to reversal learning or between reversal stages (Experiment 3 only).

## 3 Results

### 3.1 Experiment 1: evaluation of simple discrimination and reversal learning using a T-insert within a MWM

**Figure 4:**
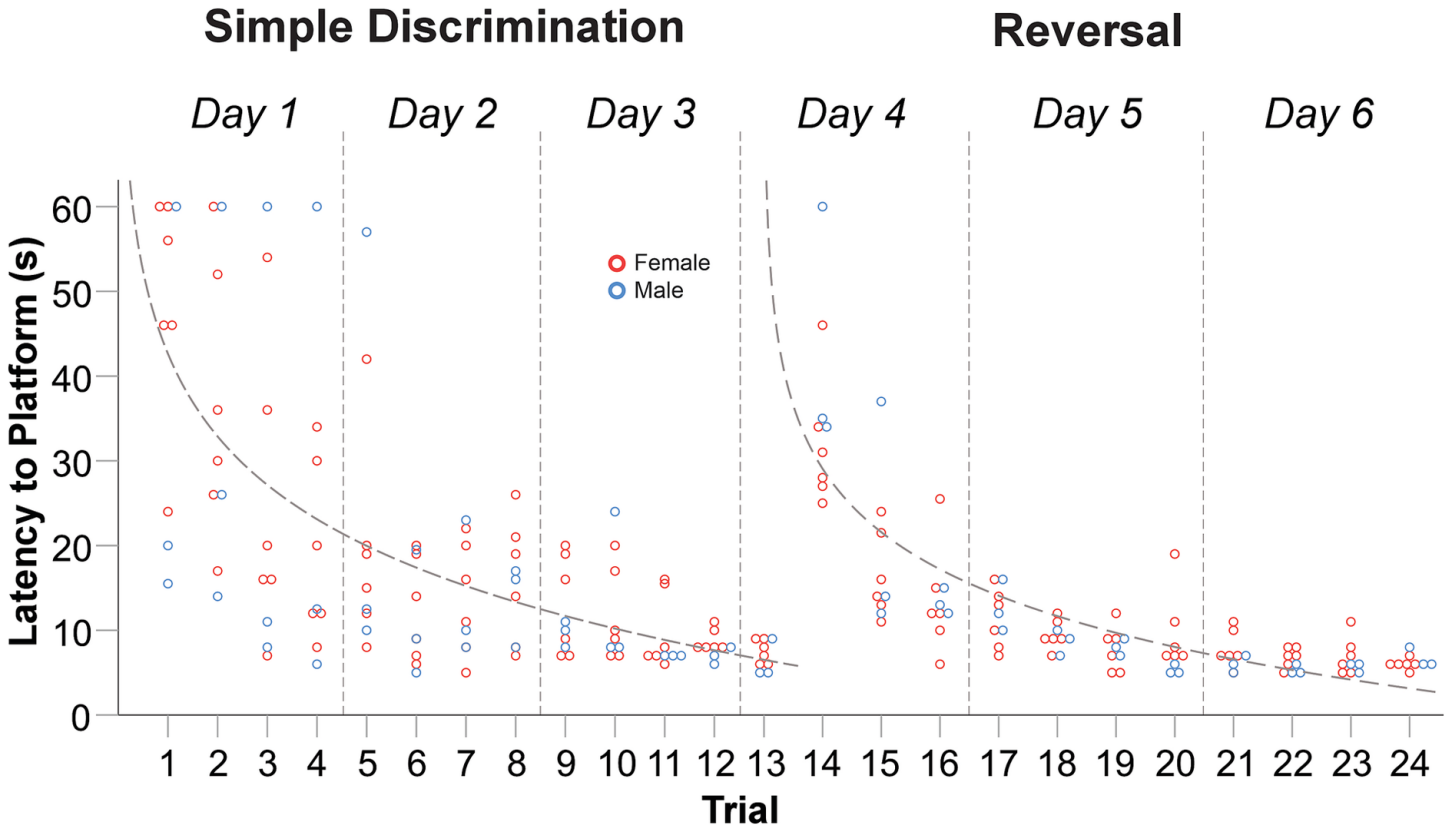
Change in latency to platform across trials, within stage, in male and female C57BL/6J mice in Experiment 1. The dashed line represents the predicted log-linear regression fitted curve. Individual data points within a trial are jittered.

Compared to the MWM, where the average trial length is ~45 s (Wahlsten, 2010b; Vorhees and Williams, 2006; Brown and Wong, 2007), the average trial length observed in our modified apparatus was notably shorter during simple discrimination [18.20 s] and reversal learning [11.87 s]. Mice therefore spent ~70% less time swimming in water close to their thermoneutral range compared to the MWM. The mean difference in latency to escape the maze between the first and last trial of simple discrimination and reversal learning was 39.74 s [*t*(8) = 8.21, *p* < 0.0001] and 29.34 s [*t*(8) = 8.33, *p* < 0.0001], respectively. This result demonstrated that animals rapidly learned to navigate to the escape platform (Figure 4). The mean difference in latency to escape between the last trial of simple discrimination and the first trial of reversal learning was −28.44 s [*t*(8) = −7.42, *p* < 0.0001], highlighting that the change in platform location elicited a search response within the apparatus. By trial 5 (mouse trial 18), however, asymptotic learning performance was already achieved, with an average escape latency of 9.22 s.

The proportion of variance (R^2^) accounted for by our log-linear regression curve fitting approach was 45.23% [*F*_1,115_ = 95.00, *p* < 0.0001, *β*_0_ = 42.63; *β*_1_ = −14.08] and 65.80% [*F*_1,97_ = 186.67, *p* < 0.0001, *β*_0_ = 29.07; *β*_1_ = −10.81] for simple discrimination and reversal learning, respectively. Thus, a good model fit and consistency with the predicted log-linear relationship typical of learning and memory paradigms was observed across both stages.

The average swim speed during simple discrimination and reversal learning was 8.57 cm/s and 7.32 cm/s, respectively. No significant differences in swim speed were observed among trials within a day, except for day 4, when the location of the escape platform was changed (between trials 13–14). Here, the average swim speed was found to be significantly different between trials 13 and 14 [*t*(8) = −2.81, *p* = 0.02], 13 and 15 [*t*(8) = −5.05, *p* = 0.001] and 14 and 15 [*t*(8) = −2.514, *p* = 0.04]. These differences were not due to thermoregulatory/waterlogging issues because swim speed increased, not decreased, when the location of the escape platform changed. Instead, these differences likely represent an increased search response that is expected when a learned contingency is changed. Altogether, these data highlight robust learning by narrowing the swimmable dimensions using a T-insert within the MWM. And, compared to the typical MWM protocol, our protocol is more efficient (6 days for the T-insert within the MWM vs. 11 days of testing for the MWM).

Given the robust learning performance observed by narrowing the swimmable area in the MWM to a “T,” we next explored whether a smaller, standalone apparatus, our WTM, would yield similarly robust data across simple discrimination and reversal learning.

### 3.2 Experiment 2: evaluation of simple discrimination and reversal learning using a standalone, dimensionally smaller, WTM

**Figure 5:**
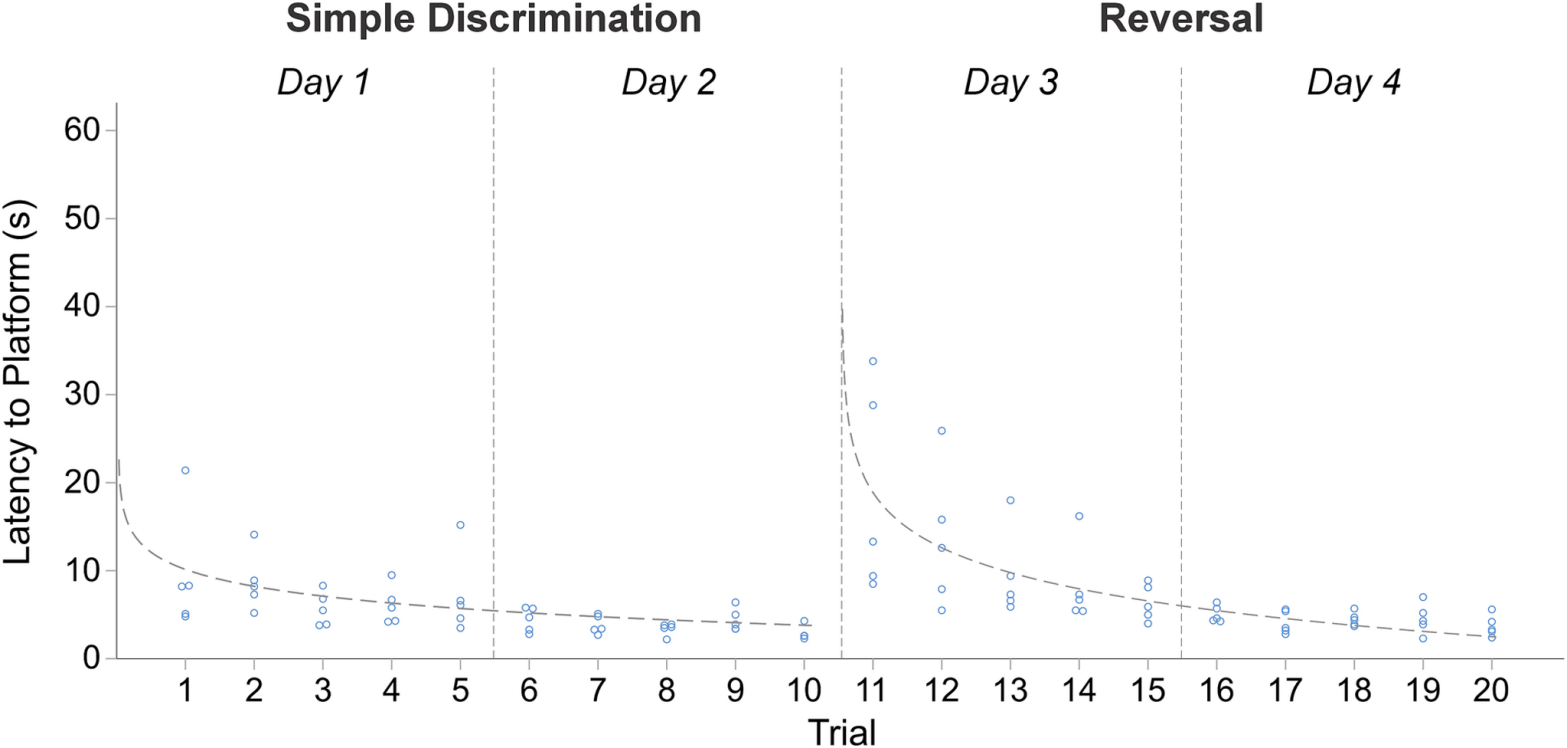
Change in latency to platform across trials, within stage, in male C57BL/6J mice in Experiment 2. The dashed line represents the predicted log-linear regression fitted curve. Individual data points within a trial are jittered.

Within our WTM, animals more rapidly learned to navigate the maze to escape from the apparatus during simple discrimination and reversal learning, compared to the larger apparatus used in Experiment 1. Consequently, mice spent less time swimming in water below their thermoneutral range compared to Experiment 1. During simple discrimination, the average latency to the escape platform on trial 1 was 9.56 s compared to 43.06 s in Experiment 1. By trial 10, the last trial of simple discrimination, the average latency to the escape platform was 2.88 s compared to 13.50 s in Experiment 1. The mean difference in latency to escape the maze between the first and last trial of simple discrimination was 6.68 s [*t*(4) = 2.33, *p* = 0.04], demonstrating that animals rapidly learned to navigate to the escape platform within the WTM. Similar patterns of behavior were observed during reversal learning; the average latency to the escape platform on trial 1 (mouse trial 11, Figure 5) of reversal learning was 18.76 s (vs. 35.56 s in Experiment 1). By the last trial of reversal learning (mouse trial 20, Figure 5), the average latency to escape was 3.72 s vs. 6.56 s in Experiment 1. The mean difference in latency to escape the maze between the first and last trial of reversal learning was 15.04 s [*t*(4) = 2.92, *p* = 0.02]. Compared to the MWM, where the average trial length is ~45 s (Wahlsten, 2010b; Brown and Wong, 2007), and to Experiment 1, the average trial length in our standalone version of the WTM was notably shorter during simple discrimination [5.62 s vs. 18.20 s] and reversal learning [7.82 s vs. 11.87 s]. The mean difference in latency to escape between the last trial of simple discrimination and the first trial of reversal learning was −15.88 s, [*t*(4) = −3.07, *p* = 0.02], highlighting that the change in platform location elicited a search response within the apparatus. By trial 3 (mouse trial 13), however, approximate asymptotic learning performance was already achieved, with an average escape latency of 9.44 s.

The proportion of variance (R^2^) accounted for by our log-linear regression curve fitting approach was 32.47% [*F*_1,48_ = 23.08, *p* < 0.0001, *β*_0_ = 9.929; *β*_1_ = −2.85] and 49.47% [*F*_1,48_ = 47.01, *p* < 0.0001, *β*_0_ = 17.77; *β*_1_ = −6.59] for simple discrimination and reversal learning, respectively. Similarly to Experiment 1, both a good model fit and consistency with the predicted log-linear relationship typical of learning and memory paradigms across both stages were observed. No significant differences in swim speed were observed between the first and last trial of testing within a day. All together these data highlight robust learning within our WTM (4 days of testing), relative to the MWM (11 days of testing) and to Experiment 1 (6 days of testing).

Given the robust learning performance observed in Experiment 2, we hypothesized that we could increase the number of trials performed per day to 10 (double that of the current experiment), while decreasing the number of test days from four to three.

### 3.3 Experiment 3: evaluation of the reliability of a modified protocol using a serial reversal learning paradigm within our WTM

**Figure 6:**
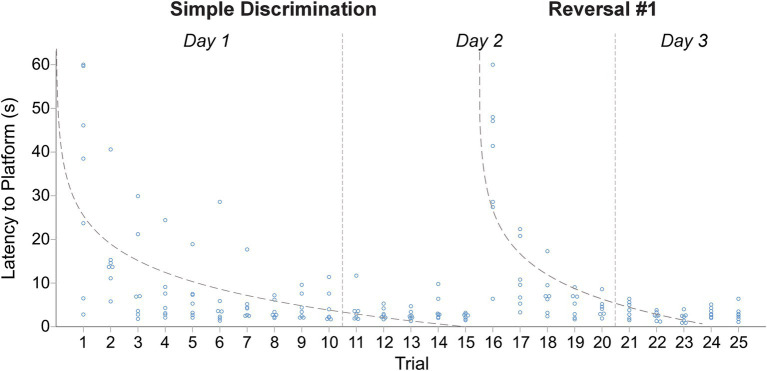
Change in latency to platform across trials, within stage, in male C57BL/6J mice in Experiment 3. The dashed line represents the predicted log-linear regression fitted curve. Individual data points within a trial are jittered.

In our modified protocol (10 vs. 5 trials per day), animals displayed robust learning across simple discrimination and reversal stages, similarly to Experiment 2, even when the number of trials per day was doubled. The mean difference in latency to escape the maze between the first and last trial was 31.51 s [*t*(6) = 3.59, *p* = 0.006] and 34.02 s [*t*(6) = 5.20, *p* = 0.001], during simple discrimination and the first incidence of reversal learning, demonstrating that animals rapidly learned to navigate to the escape platform (Figure 6). The proportion of variance (R^2^) accounted for by our log-linear regression curve fitting approach was 40.86% [*F*_1,103_ = 71.17, *p* < 0.0001, *β*_0_ = 25.14; *β*_1_ = −9.35] and 55.53% [*F*_1,68_ = 84.93, *p* < 0.0001, *β*_0_ = 26.85; *β*_1_ = −12.49] for simple discrimination and reversal learning, respectively. Similarly to Experiment 1, a good model fit and consistency with the predicted log-linear relationship typical of learning and memory paradigms were observed across both stages.

**Figure 7:**
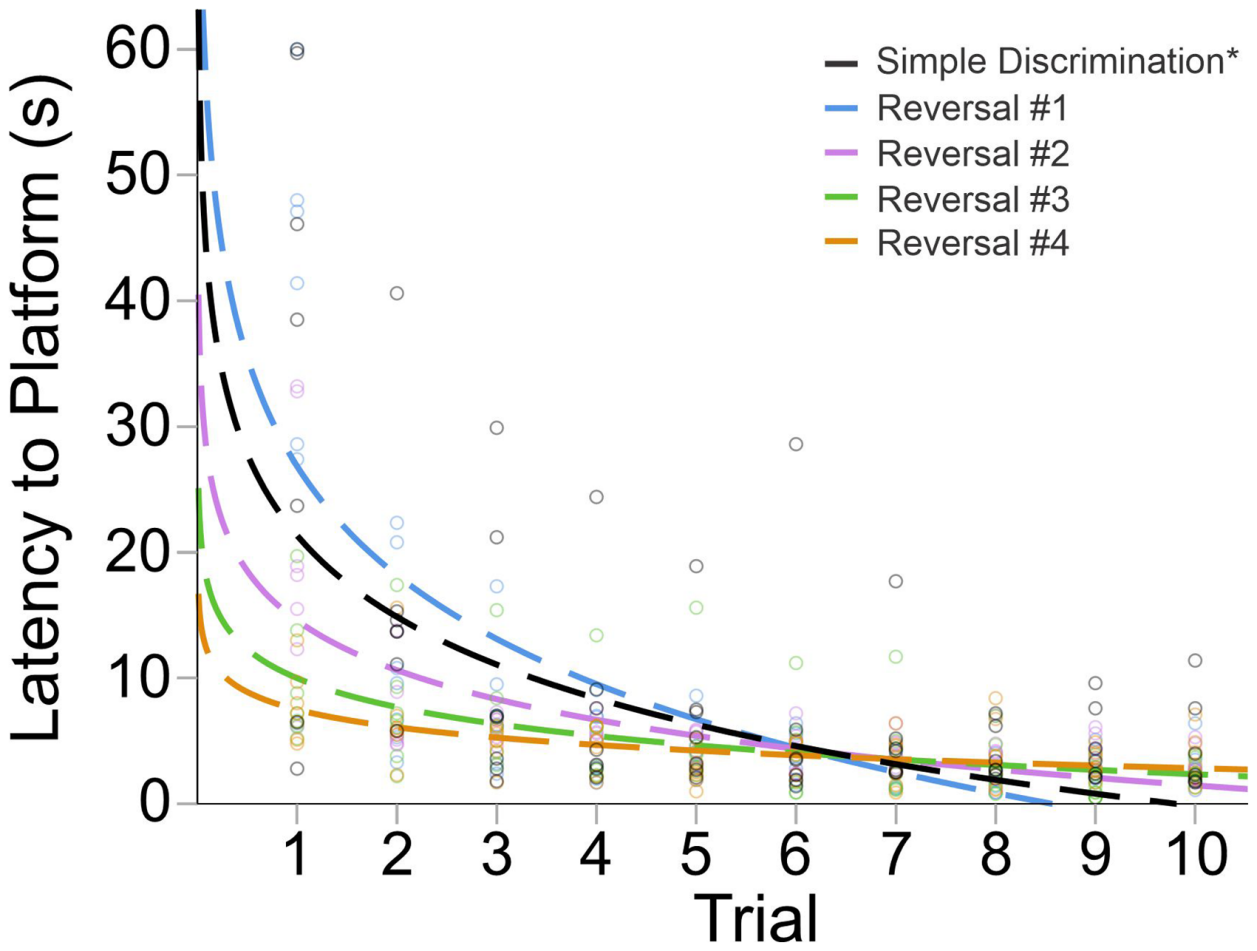
Change in latency to platform across trials and stages in Experiment 3. The dashed line represents the predicted log-linear regression fitted curve. *Subset from Figure 5; only the first 10 trials are shown.

Animals quickly updated their previously learned information when the platform was alternated to the left or right arm across stages in our serial reversal task (Figure 7). This was evidenced by a rapid decrease in mean latency to the escape platform across trials of each stage, as well as our fitted intercept and slope in our curve fitting approach (Figure 7 and Table 2). A significant difference in swim speed between the first and last trial of simple discrimination of day 1 was observed [X̄ = 5.1 cm/s, *t*(6) = −3.86, *p* = 0.004]. This result highlights that for the first day of simple discrimination only, waterlogging with a decrease in overall swim speed can occur when 10 trials are performed per day within our WTM (see Figure 6). Significant differences in swim speed were also observed between the last trial of a stage (e.g., simple discrimination) and the first trial of the subsequent stage (e.g., reversal #1). Similarly to Experiment 1, these latter differences likely represent an increased search response that is expected when a learned contingency is changed, and were not related to thermoregulatory/waterlogging issues. This conclusion was supported by an observed increase in the average swim speed when the location of the escape platform was switched [Simple Discrimination to Reversal #1 = 4.26 cm/s; Reversal #1 to Reversal #2 = 2.56 cm/s; Reversal #2 to Reversal #3 = 1.41 cm/s; Reversal # 3 to Reversal #4 = 1.05 cm/s; Reversal #4 to Reversal #5 = 1.63 cm/s]. Altogether, these data highlight robust learning using our modified protocol within our WTM (3 days of testing) relative to our previous protocol (4 days of testing), Experiment 1 (6 days of testing) and to the MWM (11 days of testing).

**Table 2:** Key descriptive statistics of latency to the escape platform across stages and from curve fitting approaches.

	Mean latency to platform			Curve fitting					
Stage	Trial 1	Trial 6	Last Trial	Intercept (*β*_0_)	Slope (*β*_1_)	df	*F*	*p*	*R* ^2^
Simple discrimination	33.90 s	6.74 s	2.39 s	25.14	−9.35	1,103	71.17	< 0.0001	40.9%
Reversal #1	36.99 s	3.85 s	2.96 s	26.85	−12.50	1,68	84.93	< 0.0001	55.5%
Reversal #2	19.57 s	4.15 s	3.24 s	14.58	−5.69	1,68	60.06	< 0.0001	46.9%
Reversal #3	9.64 s	4.20 s	3.57 s	10.00	−3.32	1,68	29.38	< 0.0001	30.2%
Reversal #4	7.76 s	3.84 s	1.87 s	7.49	−2.02	1,68	28.15	< 0.0001	29.3%

Visually, as well as quantitively, across reversals, animals achieved approximate asymptotic learning performance by ~trial 6, highlighting that the number of trials per day could be reduced to 6, while achieving the same precision of readout with respect to learning ability within our WTM (Figure 7 and Table 2). Given that we also observed a decrease in swim speed between the first and last trial of simple discrimination, likely attributable to waterlogging, we next explored the prediction that reducing the number of trials to six per day would resolve this issue, as well as yield a more efficient protocol.

### 3.4 Experiment 4: evaluation of simple discrimination and reversal learning using a truncated protocol within our WTM in a commonly used inbred and outbred strain of mouse

**Figure 8:**
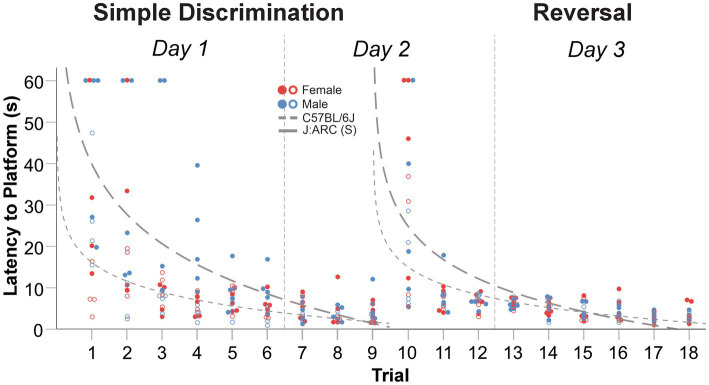
Change in latency to platform across trials, within stage, in male and female C57BL/6J and J:ARC(S) mice in Experiment 4. The dashed lines represent the predicted log-linear regression fitted curves by species. Individual data points for C57BL/6J and J:ARC(S) mice are represented by closed and open circles, respectively.

In our truncated protocol (6 vs. 10 trials per day) within our WTM, animals displayed robust learning across simple discrimination (1.5 days) and reversal stages (1.5 days), similarly to Experiments 2 and 3. The mean difference in latency to escape the maze between the first and last trial of simple discrimination was 34.41 s [*t*(8) = 5.66, *p* < 0.0001] and 14.83 s [*t*(7) = 2.96, *p* = 0.011], in C57BL/6J and J:ARC(S) mice, respectively. Similarly, the mean difference in latency to escape the maze between the first and last trial of reversal learning was 30.98 s [*t*(8) = 3.996, *p* = 0.002] and 15.19 s [*t*(7) = 3.48, *p* = 0.005], in C57BL/6J and J:ARC(S) mice, respectively. Thus, animals in both strains rapidly learned to navigate to the escape platform across both stages (Figure 8). Compared to the MWM, where the average trial length is ~45 s (Wahlsten, 2010b; Brown and Wong, 2007), the average trial length in our WTM using our truncated protocol was notably lower during simple discrimination [C57BL6/J = 14.93 s, J:ARC(S) = 6.80 s] and reversal learning [C57BL6/J = 8.43 s, J:ARC(S) = 6.33 s]. The mean difference in latency to escape between the last trial of simple discrimination and the first trial of reversal learning was −29.94 s, [*t*(8) = −3.885, *p* = 0.002] in C57BL/6J and − 14.94 s, [*t*(8) = −3.3.56, *p* = 0.005] in J:ARC(S) mice, highlighting that the change in platform location elicited a search response within the apparatus. This conclusion was supported by an overall increase in swim speed [C57BL/6J = 1.65 cm/s; J:ARC(S) = 1.86 cm/s] between the first trial of reversal learning to the last trial of simple discrimination. By the second trial, (mouse trial 11), approximate asymptotic learning performance was already achieved with an average escape latency of 7.81 s [C57BL6/J] and 4.80 s [J:ARC(S)].

The proportion of variance (R^2^) accounted for by our log-linear regression curve fitting approach was 45.3% [*F*_1,79_ = 65.452, *p* < 0.0001, *β*_0_ = 39.75; *β*_1_ = −17.45] and 41.5% [*F*_1,79_ = 56.02, *p* < 0.0001, *β*_0_ = 24.72; *β*_1_ = −11.45] for simple discrimination and reversal learning, respectively, in C57BL/6J mice. In J:ARC(S) mice, the proportion of variance (R^2^) accounted for by our log-linear regression curve fitting approach was 40.6% [*F*_1,70_ = 47.78, *p* < 0.0001, *β*_0_ = 16.07; *β*_1_ = −6.52] and 44.7% [*F*_1,70_ = 56.68, *p* < 0.0001, *β*_0_ = 14.92; *β*_1_ = −6.04] for simple discrimination and reversal learning, respectively. Thus, a good model fit and consistency with the predicted log-linear relationship typical of learning and memory paradigms were observed across both stages.

A significant difference in swim speed between the last trial of simple discrimination and the first trial of the reversal learning was observed [5.20 cm/s, *t*(8) = −3.102, *p* = 0.007]. These latter differences likely represent an increased search response that is expected when a learned contingency is changed, and were not related to thermoregulatory/waterlogging issues, as the average swim speed increased when the location of the escape platform was switched. Altogether, these data highlight robust learning using a truncated protocol within our WTM (3 days of testing/6 trials per day), relative to the MWM (11 days of testing/4 trials per day) and compared to our protocols within our WTM (4 days of testing/5 trials per day and 3 days of testing/10 trials per day). Given the robust learning observed in this version of our protocol, and what would be the final version of our WTM protocol, we next evaluated the generalizability of these observed results.

### 3.5 Experiment 5: generalizability of simple discrimination and reversal learning in the finalized WTM protocol across independent replicates and strains of mice

**Figure 9:**
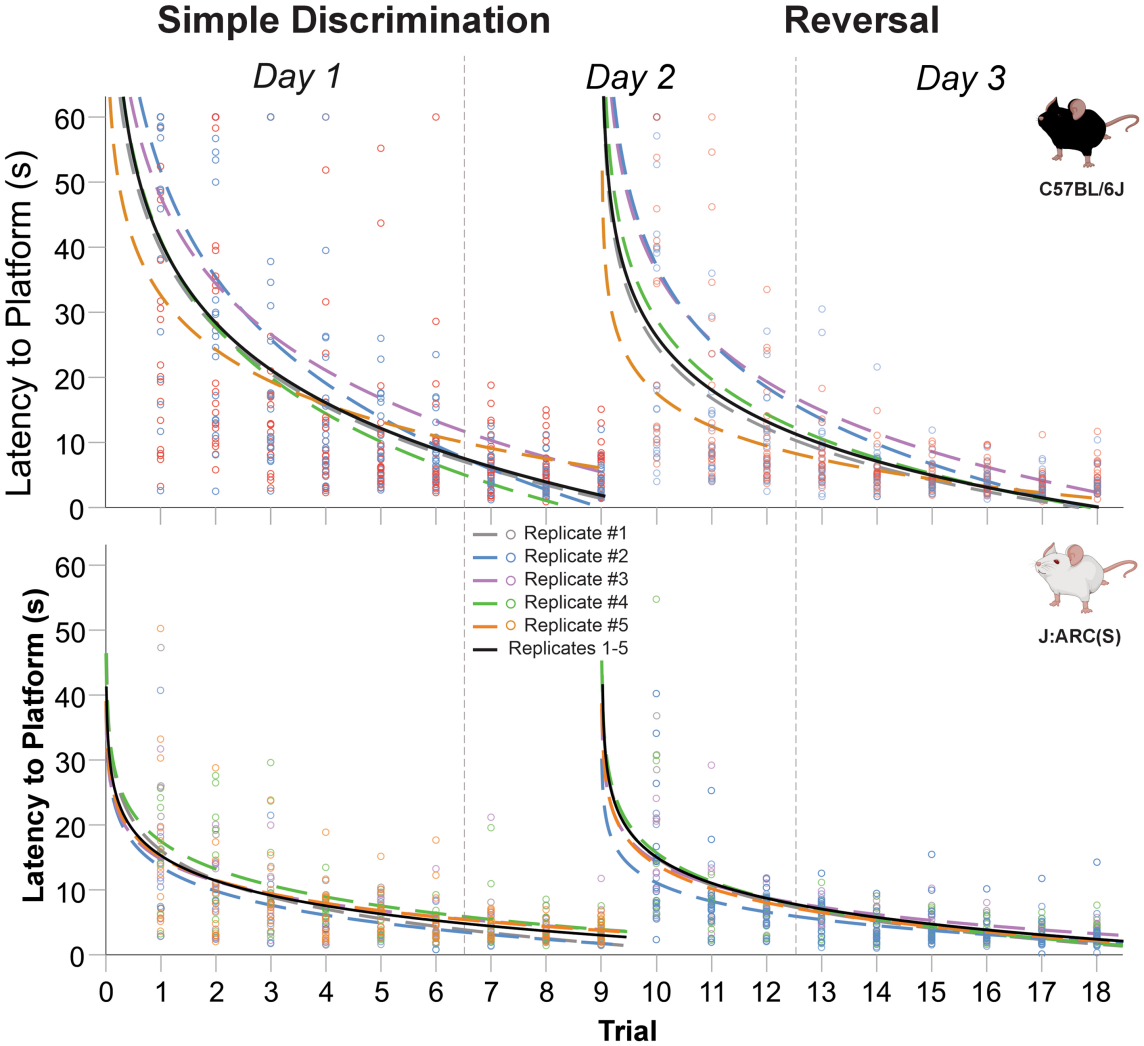
Change in latency to platform across replicates and trials, within stage, in male and female C57BL/6J and J:ARC(S) mice in Experiment 5. The dashed lines represent the predicted log-linear regression fitted curve per replicate. *Replicate 1 derived from Experiment 4.

Mice of both strains displayed a robust learning performance during simple discrimination and reversal learning, regardless of replicate (c.f., Figure 9 and Table 3). The mean difference in latency to escape the maze between the first and last trial of simple discrimination, across all replicates, was 36.02 s [*t*(44) = 11.97, *p* < 0.0001] and 11.83 s [*t*(44) = 6.00, *p* < 0.0001], in C57BL/6J and J:ARC(S) mice, respectively. Similarly, the mean difference in latency to escape the maze between the first and last trial of reversal learning, across all replicates, was 28.28 s [*t*(44) = 8.90, *p* < 0.0001] and 12.68 s [*t*(44) = 7.70, *p* < 0.0001], in C57BL/6J and J:ARC(S) mice, respectively. While there was some heterogeneity within strain and across replicates in terms of the average latency to escape the maze on the first trial of each stage, the average latency to escape the maze on the per replicate basis already approximated the overall mean latency by trial 6 (Table 3). This result highlights the robustness of our WTM protocol, where, despite initial individual differences, non-impaired animals quickly learn to navigate to the hidden platform to escape (Figure 9 and Table 3).

**Table 3:** Key descriptive statistics of latency to the escape platform across stages and from curve fitting approaches.

Stage	Replicate		Mean latency to platform			Curve fitting					
		Strain	Trial 1	Trial 6	Trial 9	Intercept (*β*_0_)	Slope (*β*_1_)	df	*F*	*p*	R^2^
Simple discrimination	Replicate #1		39.10	8.14	4.69	39.75	−17.45	1,79	65.45	< 0.0001	45.3%
	Replicate #2		57.27	8.57	6.45	51.77	−23.57	1,88	230.67	< 0.0001	72.4%
	Replicate #3	C57BL/6J	46.23	12.03	5.93	47.79	−19.26	1,34	29.54	< 0.0001	46.5%
	Replicate #4		41.91	5.33	3.16	41.18	−19.29	1,61	107.90	< 0.0001	63.9%
	Replicate #5		29.48	10.37	4.78	31.29	−12.14	1,133	53.21	< 0.0001	28.6%
	Replicates 1–5		41.00	8.89	4.98	40.54	−17.49	1,403	358.62	< 0.0001	47.1%
	Replicate #1		17.94	3.15	3.11	16.07	−6.52	1,70	47.78	< 0.0001	40.6%
	Replicate #2		15.11	3.69	2.89	13.61	−5.37	1,70	42.98	< 0.0001	38.0%
	Replicate #3	J:ARC(S)	16.50	5.73	5.46	14.80	−4.94	1,70	33.43	< 0.0001	32.3%
	Replicate #4		15.91	4.94	4.64	17.55	−6.19	1,70	37.57	< 0.0001	34.9%
	Replicate #5		17.15	6.03	6.56	15.11	−5.14	1,115	30.42	< 0.0001	20.9%
	Replicates 1–5		16.59	4.85	4.76	15.39	−5.58	1,403	173.66	< 0.0001	30.1%
Reversal	Replicate #1		34.63	4.35	3.65	24.72	−11.45	1,79	56.02	< 0.0001	41.5%
	Replicate #2		40.89	5.04	4.53	37.14	−17.01	1,88	114.07	< 0.0001	56.5%
	Replicate #3	C57BL/6J	42.08	7.15	6.93	36.30	−15.47	1,34	36.02	< 0.0001	51.4%
	Replicate #4		37.20	4.40	4.30	28.85	−13.21	1,61	58.68	< 0.0001	49.0%
	Replicate #5		20.67	4.02	3.40	17.55	−7.35	1,133	72.06	< 0.0001	35.1%
	Replicates 1–5		32.43	4.65	4.15	26.76	−11.95	1,403	291.99	< 0.0001	42.0%
	Replicate #1		18.05	3.58	2.86	14.92	−6.04	1,70	56.68	< 0.0001	44.7%
	Replicate #2		11.83	3.44	2.93	11.15	−4.10	1,70	61.74	< 0.0001	46.9%
	Replicate #3	J:ARC(S)	14.85	5.88	4.23	14.38	−5.04	1,70	61.68	< 0.0001	46.8%
	Replicate #4		20.84	4.11	4.20	15.77	−6.34	1,70	33.13	< 0.0001	32.1%
	Replicate #5		15.94	4.95	3.65	13.93	−5.32	1,115	62.03	< 0.0001	35.0%
	Replicates 1–5		16.26	4.45	3.58	14.02	−5.36	1,403	240.98	< 0.0001	37.4%

The average proportion of variance (R^2^) accounted for by our log-linear regression curve fitting approach was 47.1% [*F*_1,403_ = 358.62, *p* < 0.0001, *β*_0_ = 40.54; *β*_1_ = −17.49] and 42.0% [*F*_1,403_ = 291.99, *p* < 0.0001, *β*_0_ = 26.76; *β*_1_ = −11.95] for simple discrimination and reversal learning, respectively, in C57BL/6J mice. In J:ARC(S) mice, the proportion of variance (R^2^) accounted for by our log-linear regression curve fitting approach was 30.1% [*F*_1,403_ = 173.66, *p* < 0.0001, *β*_0_ = 15.39; *β*_1_ = −5.58] and 37.4% [*F*_1,403_ = 240.98, *p* < 0.0001, *β*_0_ = 14.02; *β*_1_ = −5.36] for simple discrimination and reversal learning, respectively. Similar patterns were observed within strain and across replicates and stage (c.f., Table 3). Thus, a good model fit and consistency with the predicted log-linear relationship typical of learning and memory paradigms, across both stages, were observed.

Significant differences in swim speed were also observed between the last trial of simple discrimination and the first trial of reversal learning. These differences likely represent an increased search response that is expected when a learned contingency is changed, and were not related to thermoregulatory/waterlogging issues, as the average swim speed increased when the location of the escape platform was switched [C57BL/6J; Replicate 1 = 5.19 cm/s; Replicate 2 = 1.94 cm/s; Replicate 3 = 1.51 cm/s; Replicate 4 = 4.31 cm/s; Replicate 5 = 1.65 cm/s and in J:ARC(S); Replicate 1 = 1.86 cm/s; Replicate 2 = 2.67 cm/s; Replicate 3 = 0.85 cm/s; Replicate 4 = 0.93 cm/s; Replicate 5 = 2.13 cm/s]. Altogether, these data highlight robust learning using our finalized protocol within our WTM (3 days of testing, 6 trials per day), relative to the MWM (11 days of testing, 4 trials per day) and compared to our previous protocols within our WTM (4 days of testing, 5 trials per day and 3 days of testing at 10 trials per day). Given the robust learning observed in the final version of our WTM protocol, we next explored the prediction that mice used spatial strategies to navigate to the escape platform.

### 3.6 Experiment 6: discriminating between the use of taxis vs. spatial strategies in our finalized WTM protocol

In Experiment 5, during the last three trials of reversal learning (i.e., trials 16–18), 100% of all animals tested in both strains immediately swam to the escape platform (located in the right arm) when released from the start arm (Figure 4A). When animals were released from the left arm on trial 19, instead of the start arm, 44 out of 45 C57BL/6J mice [97.78%] and 45 out of 45 J:ARC(S) mice [100%] swam immediately to the escape platform located in the right arm (i.e., straight ahead, Figure 4B). In trial 20, when animals were again released from the start arm, all 45 C57BL/6J and J:ARC(S) mice [100%] swam immediately to the escape platform located in the right arm (i.e., straight ahead and then immediately turned right, Figure 4C). No statistical analyses were performed here, as the variance in these results was essentially zero. Together, these results highlight that the animals used distal cues to rapidly navigate to the hidden escape platform within our WTM.

## 4 Discussion

The overarching goals of this study were to establish our novel WTM apparatus and associated protocol as an efficient initial screening tool for learning, memory and executive function behaviors in laboratory mice. To accomplish these goals, we also incorporated a number of important factors into our testing procedures which have been shown to improve animal welfare, as well as performance and measurement of behavior in similar water-based assays: reduction of thermoregulatory distress, animal handling, and infrared backlighting (Bailoo et al., 2010; Iivonen et al., 2003; Roder et al., 1996; Gouveia and Hurst, 2013; Gouveia and Hurst, 2017; Gouveia and Hurst, 2019). As an initial proof of principle, we first narrowed the swimmable area within the MWM to a “T” and demonstrated that animals quickly learned to spatially navigate to the hidden escape platform during simple discrimination and reversal learning. By further narrowing the swimmable area by 43% in our standalone WTM, we demonstrated similar levels of robust learning but in a more efficient protocol. Our finalized protocol, requiring 3 days of testing, is also significantly shorter than the 11 days required for testing to obtain similar kinds of data within the MWM; animals therefore spend less time swimming in water below their thermoneutral range, which correspondingly results in shorter daily test sessions and lower levels of stress. Moreover, by constraining the swimmable area of the apparatus to a “T,” off-task behavior was eliminated in all experiments with our WTM.

Our WTM apparatus and protocol solve one of the more contentious issues with the MWM—the need for a probe trial to evaluate the strength of the learned response after a fixed number of training trials have been performed (Wahlsten, 2010b; Wahlsten et al., 2005). Animals are placed within the MWM at locations that are not equidistant from the escape platform, and animals rarely swim in a straight line toward the escape platform. As a consequence, a data smoothing approach is used, where, for example, the average latency to the escape platform within a day is calculated. Thus, inference about the learning ability of mice within the MWM is difficult, if not impossible. In contrast, within our WTM, animals are always started from the same location and consequentially quickly learn to swim directly to the hidden escape platform. As such, there is no need to disambiguate a bewildering variety of complex paths that often vary within and between mice, typical of the MWM (Wolfer et al., 1998; Dalm et al., 2000).

Both the MWM and our WTM require that the animal extinguish its previously learned response during reversal learning. It can potentially be argued that this extinction process may be more complex within the MWM due to the variable start locations that are used within and between test sessions and the re-mapping of the previously acquired spatial-map within this context. While this may well be true, empirically addressing this question would require data from a large number of subjects. As far as we are aware, only one paper has systematically addressed this question by analyzing data from 1,400 mice and over 50,000 swim tracks (Wolfer et al., 1998). In that paper, the authors demonstrated that thigmotaxis and passivity accounted for 68% of the explained variance in the data while memory accounted for a mere 19% of the explained variation. The authors thus concluded that “more than two-thirds of the behavioral variability is accounted for by two factors that have no direct relation to spatial memory and learning.....” These data and conclusions are important because they fundamentally challenge the notion that the MWM protocol is a robust tool for the evaluation of spatial learning and memory. Correspondingly, these data challenge the assumption that reversal learning in the MWM is a more complex process than within our WTM, as thigmotaxis and passivity are not observed within our apparatus and protocol.

While WTMs for mice have been previously described in the literature, they vary unsystematically in apparatus dimensions and fail to establish key aspects of internal and external validity in their associated protocols; none of those studies have evaluated simple discrimination and reversal learning similar to the standard MWM protocol, which is the focus of our study (Cahill et al., 2015; Davis et al., 2017; Guariglia and Chadman, 2013; Wainwright et al., 1990). We demonstrated that our WTM apparatus and protocol show good internal validity and external validity; animals used distal cues to navigate to the hidden escape platform (Experiment 6), while behavioral performance in our WTM apparatus and associated protocol generalized to commonly used strains of mice, both sexes, ages, replicates, and even across minor procedural differences (Experiments 2–5). Methodologically, setup time was also more efficient as the volume of water that is filled, drained and heated is 91% smaller compared to a 120 cm diameter MWM filled to a height of 11 cm.

Non-water-based T-maze protocols have also previously been described in the literature, primarily for the assessment of spatial working memory-related, rewarded- or spontaneous- alternation behavior (Wenk, 1998; Spowart-Manning and van der Staay, 2004; Shoji et al., 2012; Deacon and Rawlins, 2006; d'Isa et al., 2021). Rewarded versions of that protocol are likely stressful because it involves long periods of food restriction to 85–90% the free feeding body weight (Deacon and Rawlins, 2006). In contrast, spontaneous alternation versions of the protocol, that do not involve food restriction, may be limited in their precision as only 5–6 trials are performed (d'Isa et al., 2021). For example, in one common protocol of spontaneous alternation, the authors concluded that the chance level for spatial working memory in a two-choice discrimination task within a T-maze was 50% (d'Isa et al., 2021); this threshold can only be considered accurate if each trial performed by a single mouse was considered independent from previous trials. In this particular example, only the first trial/arm entry and the second trial/arm entry (if the choice is the previously unexplored arm), can only be reasonably considered independent events (Dember and Richman, 2012; Manning, 1973a; Manning, 1973b). Combining this issue with the expected results data which show that, on average, mice spontaneously alternate for only four of the five or six presented trials, calls into question the validity of the spontaneous alternation protocol (c.f., Figure 2) (d'Isa et al., 2021). More importantly, however, is that these kinds of data would require significantly more trials to satisfy the assumptions of the appropriate statistical model [e.g., linear mixed effects modeling with a binomial error distribution, Markov Chain analyses etc. (Bailoo et al., 2018; Bailoo et al., 2018; Bailoo et al., 2020; Gygax, 2014)]. These issues, coupled with a potential for off-task behavior in the absence of a motivating stimulus (c.f., sections on Troubleshooting and Advantages and Limitations, d'Isa et al., 2021), may make evaluation of data from a spontaneous alteration task questionable. Altogether, these issues with the non-water-based T-maze protocols, make our WTM apparatus and protocol an important novel alternative for screening spatial-related learning and memory impairments in laboratory mice.

Standardization of behavioral protocols has been repeatedly shown to improve reliability and reproducibility of derived experimental results, both across laboratories of the same investigator (Wahlsten et al., 2006) and across laboratories with different investigators (Arroyo-Araujo et al., 2022). Unlike the MWM, where protocols vary in unsystematic ways (Wahlsten, 2010b; Kapadia et al., 2016), our WTM apparatus and protocol are robust against such issues, given the simplicity of the apparatus’ design and associated protocol. Moreover, the small amounts of space and associated infrastructure that are needed for its implementation renders it amenable to a high degree of standardization across a myriad of laboratory settings.

It is important to note that no single test of learning and memory is sufficient to understand the complexity of such phenomena, particularly in a diseased state. We therefore advocate for the use of our WTM apparatus and protocol, as an initial screening tool, in tandem with other behavioral tasks (e.g., operant tasks) which evaluate the same constructs (e.g., learning, memory, executive functioning). It is important to also understand how other tasks might be limited and the consequences of their associated procedures on the behavior under consideration. For example, operant tasks often require food restriction in order to motivate the animal to respond. Food restriction has a number of consequences on interoceptive states as well as with respect to behavioral readouts; impulsive and behavioral inflexibility is commonly observed using those tasks due to hunger/satiety (Bailoo et al., 2018; Wahlsten, 2010a; Bailoo et al., 2018; Bailoo et al., 2020; Harshaw, 2015; Wilcox et al., 2024). With that being said, testing mice using our WTM apparatus and protocol as well as with, for example, a two-choice discrimination nose-poke initiated operant task, tells us more about the learning ability of mice than does either task by itself.

As an initial screening tool, we found that our WTM was sensitive enough to detect cognitive impairments in mouse models of Alzheimer’s disease, alternative polyadenylation, and where key brain transporters and/or proteins have been knocked out (data not shown). As with any screening tool, we recommend further interrogating the strength and specificity of these differences using other behavioral tasks within the domain of cognition. In our case, we are further evaluating some of these initially observed differences using attention set-shifting, judgment bias, gambling tasks, fixed and variable ratios tasks, etc. using operant conditioning.

Learning spatial relationships, as required by water-based assays such as the MWM and our WTM, often requires the suppression of competing tendencies, e.g., thigmotaxis, thermoregulation. If those tendencies overwhelm the behavioral and physiological capacities of the animal, then the interpretation of the correspondingly observed behavior is contentious. Our WTM apparatus and associated protocol minimize, if not completely eliminate, the most problematic tendencies (off-task behavior) observed within the MWM. We therefore conclude that our overarching goals, to establish our novel WTM apparatus and associated protocol as an efficient assay of learning, memory and executive function behaviors in laboratory mice, were met.

## Data Availability

The data in this paper are derived from control/wildtype animals used in ongoing projects. Thus, the raw data supporting the conclusions of this article will be made available by the authors, without undue reservation, for the purpose of reproducing the described experimental results.
